# Predictors of post-intubation hypotension in trauma patients following prehospital emergency anaesthesia: a multi-centre observational study

**DOI:** 10.1186/s13049-023-01091-z

**Published:** 2023-06-02

**Authors:** James Price, Lyle Moncur, Kate Lachowycz, Rob Major, Liam Sagi, Sarah McLachlan, Chris Keeliher, Alistair Steel, Peter B. Sherren, Ed B. G. Barnard

**Affiliations:** 1 Department of Research, Audit, Innovation, and Development, East Anglian Air Ambulance, Norwich, UK; 2 Emergency Department, Cambridge University Hospitals NHS Foundation Trust, Cambridge, UK; 3 Essex and Herts Air Ambulance, Earls Colne, UK; 4 Anglia Ruskin University, Chelmsford, UK; 5 Magpas Air Ambulance, Huntingdon, UK; 6 Department of Critical Care Medicine, Guy’s and St Thomas’ NHS Foundation Trust, London, UK; 7 Academic Department of Military Emergency Medicine, Royal Centre for Defence Medicine (Research & Clinical Innovation), Birmingham, UK

**Keywords:** Hypotension, Post-intubation hypotension, Prehospital, Prehospital emergency anaesthesia, Rapid sequence induction

## Abstract

**Background:**

Post-intubation hypotension (PIH) after prehospital emergency anaesthesia (PHEA) is prevalent and associated with increased mortality in trauma patients. The objective of this study was to compare the differential determinants of PIH in adult trauma patients undergoing PHEA.

**Methods:**

This multi-centre retrospective observational study was performed across three Helicopter Emergency Medical Services (HEMS) in the UK. Consecutive sampling of trauma patients who underwent PHEA using a fentanyl, ketamine, rocuronium drug regime were included, 2015–2020. Hypotension was defined as a new systolic blood pressure (SBP) < 90 mmHg within 10 min of induction, or > 10% reduction if SBP was < 90 mmHg before induction. A purposeful selection logistic regression model was used to determine pre-PHEA variables associated with PIH.

**Results:**

During the study period 21,848 patients were attended, and 1,583 trauma patients underwent PHEA. The final analysis included 998 patients. 218 (21.8%) patients had one or more episode(s) of hypotension ≤ 10 min of induction. Patients > 55 years old; pre-PHEA tachycardia; multi-system injuries; and intravenous crystalloid administration before arrival of the HEMS team were the variables significantly associated with PIH. Induction drug regimes in which fentanyl was omitted (0:1:1 and 0:0:1 (rocuronium-only)) were the determinants with the largest effect sizes associated with hypotension.

**Conclusion:**

The variables significantly associated with PIH only account for a small proportion of the observed outcome. Clinician gestalt and provider intuition is likely to be the strongest predictor of PIH, suggested by the choice of a reduced dose induction and/or the omission of fentanyl during the anaesthetic for patients perceived to be at highest risk.

## Background

In the United Kingdom (UK), a significant proportion of the most seriously injured trauma patients have airway compromise requiring intervention that exceeds the capabilities of the statutory ambulance service [1]. UK Helicopter Emergency Medical Services (HEMS) deliver prehospital emergency anaesthesia (PHEA) more than two-thousand times per year, predominantly in patients with traumatic head injury, where meticulous avoidance of hypoxia and hypotension are key to reducing secondary brain injury and improving outcomes [2, 3].

Drug-assisted rapid sequence induction (RSI) is used to facilitate prehospital emergency intubation [4, 5]. Simple, standardised RSI protocols are recommended for PHEA to promote reproducible techniques and reduce human error [6]. The historical use of etomidate and suxamethonium has been superseded by fentanyl, ketamine, and rocuronium administered either in a full dose (3:2:1) or reduced dose (1:1:1) regime [7]. These protocols yield favourable intubating conditions [8], but the addition of an opioid may increase the risk of post-intubation hypotension (PIH) [9–11]. PIH after PHEA is prevalent, and is associated with increased mortality in trauma [12–14]. Therefore, prehospital key performance indicators include the incidence of PIH as a marker of quality in UK HEMS practice [15, 16].

The factors associated with PIH in critically injured patients are not well understood but are likely to be a combination of haemorrhage, cardiac depression from contusions and/or hypoxia, negative inotropy and vasoplegia from anaesthetic agents, acidaemia secondary to hypercapnia during peri-intubation apnoea, and reduced venous return from positive-pressure ventilation. Previous publications on this topic include small sample sizes with an inherent inability to reliably characterise the determinants of PIH [17, 18]. The objective of this multi-centre observational study was to compare the differential determinants of PIH in a large cohort of undifferentiated adult trauma patients.

## Methods

### Setting

The study was performed in three UK HEMS with five operational bases: two are operated by East Anglian Air Ambulance (EAAA), two by Essex & Herts Air Ambulance (EHAAT), and one by Magpas Air Ambulance (Magpas). HEMS provide prehospital critical care on behalf of the statutory ambulance service in the East of England (The East of England Ambulance Service NHS Trust (EEAST)) to a population of over six million people over a geographic area of 20,000 km^2^ [19], dispatched by either rotary wing (H145 (EAAA), AW169 (EHAAT/Magpas), or MD902 (EHAAT)) or rapid response vehicle, depending on patient location, weather constraints, and time of day.

The core of each team consists of a physician and a critical care paramedic with at least three years’ post-registration experience. Physicians in these teams are predominantly emergency medicine (EM) or anaesthesia consultants or senior registrars (at least five years post-registration), with a minimum of six months training in hospital anaesthesia. Prior to independent practice, physicians undergo further specialist training in prehospital care, including a period of supervision and local formative assessment prior to independent practice [8].

These services deliver PHEA according to a shared guideline [8]. This includes a standardised drug regime: ketamine (1–2 mg kg^−1^), rocuronium (1 mg kg^−1^), ± fentanyl (1–3 mcg kg^−1^) at the discretion of the attending clinician to attenuate the hypertensive response to laryngoscopy; subjectively tailored to each patient, based on factors such as age and haemodynamics [7]. Intubation is typically performed using direct laryngoscopy. In 2017, the option (for use at the discretion of the attending clinician) of videolaryngoscopy was introduced at EAAA and Magpas (McGrath® videolaryngoscope, Aircraft Medical, Edinburgh, UK). All services use positive pressure ventilation targeting a tidal volume of 7 ml kg^−1^ (ideal body weight) with an initial PEEP of 5 cm H_2_O and a frequency set to achieve normocapnia. A pre-induction checklist attempts to identify and initiate correction of physiological derangement prior to administering anaesthesia. All services use HEMSbase (MedicOne Systems Ltd, UK) electronic medical record software.

### Inclusion criteria

In this retrospective observational study, a consecutive sample of trauma patients ≥ 16 years old, attended to by EAAA or EHAAT (1st January 2015 to 31st December 2020) or Magpas (1st November 2015 to 31st December 2020, owing to later HEMSbase implementation) and underwent PHEA were included.

### Exclusion criteria

Clinical records were reviewed by one of the study authors to identify exclusions: duplicate cases, unascertainable patient age, secondary transfer, intubated in arrest, intubation by a non-HEMS clinician, and mechanisms not meeting the definition of trauma (injury through the transfer of kinetic energy); including medical cases initially coded as ‘trauma’, overdose, hanging, asphyxiation, burns, drowning, electrocution. Records were also excluded if systolic blood pressure readings were not available pre- and post-PHEA.

### Data collection

Anonymised data were extracted from HEMSbase and collated into a password-protected data sheet (Microsoft® Excel for Mac, v16.45) stored on a secure server.

The following data items were retrieved: demographics (age, sex, estimated weight), trauma type (blunt or penetrating), mechanism of injury, Glasgow Coma Scale (GCS) score, injury pattern suspected by attending clinician, indication for PHEA, time interval from arrival of HEMS team to PHEA, and intravenous crystalloid administration by EEAST before HEMS arrival.

Physiological variables were captured from time-calibrated patient monitors (EAAA – X Series, ZOLL Medical Corporation, Runcorn, UK; EHAAT & Magpas – Tempus Pro, Philips Electronics UK Ltd, Farnborough, UK) and uploaded automatically to HEMSbase at two-minute (EAAA, EHAAT) or three-minute (Magpas) intervals. Using manual review of each case by the study authors, the following pre-PHEA physiological variables were recorded based on the closest time-point preceding the recorded time of PHEA: heart rate (HR), respiratory rate (RR), systolic blood pressure (SBP), diastolic blood pressure (DBP), and derived shock index (SI). Post-PHEA SBP readings were recorded at the time points closest to two, four, six, eight, and ten-minutes post-PHEA. Data were excluded if deemed explicitly erroneous. If data were equivocal, a decision to include was reached by consensus after independent review of all available case notes.

PHEA drug doses of fentanyl (mcg kg^−1^), ketamine (mg kg^−1^), and rocuronium (mg kg^−1^), were calculated using the recorded dose and estimated patient weight. These were rounded to the nearest integer and then summarised by the universally cited regimes of drug administration (fentanyl:ketamine:rocuronium) e.g., 3:2:1, 1:1:1 etc. Records of patients who had been administered a vasoactive drug (metaraminol, ephedrine, or adrenaline) were individually reviewed to record the time of vasopressor administration and coded as pre-RSI, post-RSI ≤ 10 min, post-RSI > 10 min, and post-RSI < time unknown > .

### Outcome measure

Hypotension was defined as a new SBP < 90 mmHg ≤ 10 min of induction, or a > 10% drop if SBP was < 90 mmHg pre-PHEA [20].

### Data analysis

Data manipulation and statistical analyses were performed using the R statistical programming language (R Core Team [2018]; R: A language and environment for statistical computing [R Foundation for Statistical Computing, Vienna, Austria]). Statistical significance was pre-defined as *p* < 0.05. Characteristics of the sample were described as number (percentage) for categorical variables, and mean (± standard deviation (SD)) or median [interquartile range (IQR)] for continuous variables as appropriate.

A purposeful selection logistic regression model was used [21]. Each variable was first tested in turn to explore the unadjusted association with the outcome. Variables with a *p*-value < 0.25 and variables of known clinical importance were included in the multivariable analysis. Variables were then sequentially eliminated until only statistically significant variables remained and the model achieved the best fit based on likelihood ratio tests. The assumptions of logistic regression were tested, checking for linear relationships in the logit of the outcomes, unduly influential values and multicollinearity. Plausible interactions were tested, with likelihood ratio tests and McFadden’s pseudo R-squared used to determine the final best model.

Administration of vasoactive medication pre-PHEA was not defined a-priori as a determinant of hypotension. Therefore, vasopressor administration was not included in the purposeful model build. In recognition that post-PHEA vasopressor administration could confound the results or attenuate the outcome, the sensitivity of the final model to including vasopressor administration was evaluated.

For categorical variables, the group containing the largest number of cases was used as the reference group in the regression model. Patients were divided into four age groups. Pre-PHEA SBP was grouped as: Low (< 90 mmHg), Mid (90–140 mmHg), High (> 140 mmHg), heart rate was grouped as: Low (< 60 beats/min), Mid (60–100 beats/min), High (> 100 beats/min), and respiratory rate was grouped as: Low (< 10 breaths/min), Mid (10–25 breaths/min), High (> 25 breaths/min) [22]. For drug regimes, the 3:2:1 dose regime was used as the reference group, compared with 1:1:1, 0:1:1, and 0:0:1 (rocuronium only); alternative regimes were coded as ‘other’ [7].

### Ethical review

Ethical approval for the study was granted by Anglia Ruskin University Research Ethics Panel (AH-SREP-20-047). The study was registered and approved by each participating organisation. The STROBE (Strengthening the Reporting of Observational studies in Epidemiology) reporting guideline was followed [23].

## Results

**Fig. 1:**
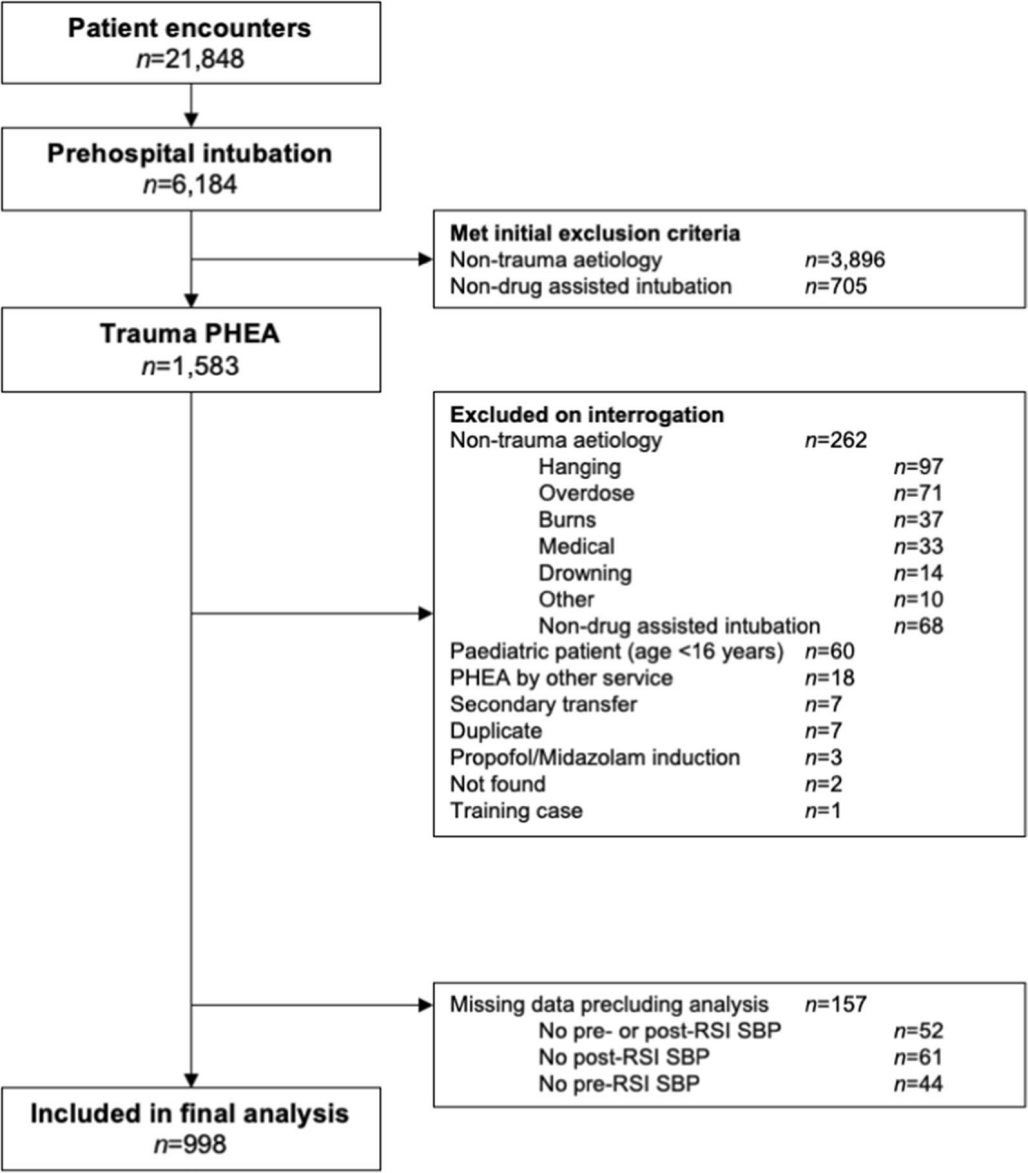
Adult trauma patients who underwent PHEA in the East of England, 2015–2020. Study flow diagram. Cases excluded on interrogation based on mechanism of injury with *n* ≤ 10 are grouped to ‘Other’ to protect patient confidentiality and include: smoke inhalation, asphyxiation, electrocution, and hypothermia. ‘Training case’ refers to fictional patient record that was created for the purpose of training and education. PHEA prehospital emergency anaesthetic, RSI rapid sequence induction, SBP systolic blood pressure

During the study period 21,848 patients were attended by HEMS, and 1,583 trauma patients underwent PHEA. 998 cases were included in the final analysis: EHAAT *n* = 426 (42.7%), EAAA *n* = 416 (41.7%), Magpas *n* = 156 (15.6%), Fig. 1.

The median average time to PHEA from the initial emergency call was 58 [48–71] minutes. Most injuries resulted from blunt trauma, and ‘transport’ was the most prevalent mechanism of injury. Suspected isolated head injury was the most frequently observed injury pattern. The median pre-PHEA SI was 0.71 [0.55–0.95], and *n* = 112 (11.2%) patients had a pre-PHEA SBP < 90 mmHg. The most prevalent drug regime was 3:2:1. The most common PHEA indication was ‘reduced consciousness’, and just over a quarter of patients were administered intravenous crystalloid before HEMS arrival, Table 1.

**Table 1:** Patient characteristics, physiological variables and PHEA characteristics in adult trauma patients who underwent PHEA in the East of England, 2015–2020, *n* = 998

Variable	Total *n* (%)
Sex/n (%)	
Male	753 (75.5%)
Female	245 (24.5%)
Age group/n (%)	
16–34	328 (32.9%)
35–54	298 (29.9%)
55–74	254 (25.5%)
75 +	118 (11.8%)
Estimated patient weight/kg, median [IQR]	80 [70–80]
GCS score/median [IQR]	7 [4–12]
Suspected injury pattern/n (%)	
Isolated head injury	510 (51.1%)
Head injury + thorax/abdomen	354 (35.5%)
No head injury	134 (13.4%)
Trauma type/n (%)	
Blunt	964 (96.6%)
Penetrating	34 (3.4%)
Mechanism/n (%)	
Transport	562 (56.3%)
Accidental Injury	313 (31.4%)
Assault	50 (5.0%)
Self-harm	42 (4.2%)
Sport/leisure	31 (3.1%)
Shock index/median [IQR]	0.71 [0.55–0.95]
Pre-PHEA SBP/mmHg, n (%)	
Low < 90)	112 (11.2%)
Mid (90–140)	491 (49.2%)
High (> 140)	395 (39.6%)
Pre-PHEA HR/beats/min, n (%)	
Low (< 60)	84 (8.4%)
Mid (60–100)	468 (46.9%)
High (> 100)	434 (43.5%)
NA	12 (1.2%)
Pre-PHEA RR/breaths/min, n (%)	
Low (< 10)	59 (5.9%)
Mid (10–25)	458 (45.9%)
High (> 25)	257 (25.8%)
NA	224 (22.4%)
PHEA drug regime/n (%)	
3:2:1	303 (30.4%)
1:1:1	238 (23.9%)
0:1:1	214 (21.4%)
Rocuronium only	44 (4.4%)
Other	199 (19.9%)
Indication for PHEA/n (%)	
Reduced consciousness	435 (43.6%)
Airway obstruction/compromise	204 (20.4%)
Ventilatory failure	131 (13.1%)
Agitated head injury	125 (12.5%)
Anticipated clinical course	90 (9.0%)
Other	13 (1.3%)
Arrival time to PHEA in minutes/median [IQR]	22 [16–30]
Pre-PHEA fluids/n (%)	
None	712 (71.3%)
Fluids	286 (28.7%)
Vasopressor use/n (%)	
Not given	899 (90.1%)
Pre RSI	6 (0.6%)
Post RSI ≤ 10 min	21 (2.1%)
Post RSI > 10 min	46 (4.6%)
Post RSI-time unknown	26 (2.6%)

The shock index was calculated as HR/SBP. ‘Arrival to PHEA’ is the time in minutes from the HEMS team arrival on scene until the time of PHEA. Pre-PHEA fluids are intravenous crystalloid administration by the ambulance service before arrival of HEMS

*PHEA* prehospital emergency anaesthetic, *GCS* Glasgow Coma Scale, *RSI* rapid sequence induction, *HR* heart rate, *SBP* systolic blood pressure, *RR* respiratory rate, *NA* data not available

**Fig. 2:**
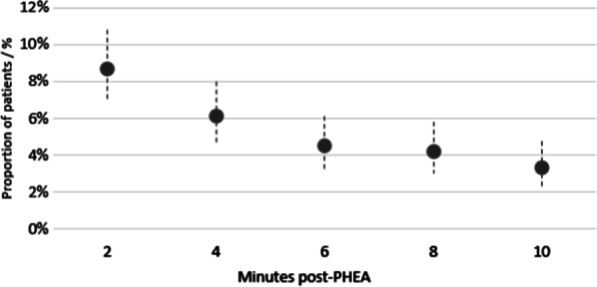
Point estimate chart showing the proportion of adult trauma patients who underwent PHEA in the East of England with a new episode of hypotension (defined as a new SBP < 90 mmHg ≤ 10 min of induction, or a > 10% drop if SBP was < 90 mmHg pre-PHEA) at two-minute epochs within the first ten minutes following induction. 95% Confidence Intervals, Wilson Score Method. PHEA prehospital emergency anaesthetic

**Fig. 3:**
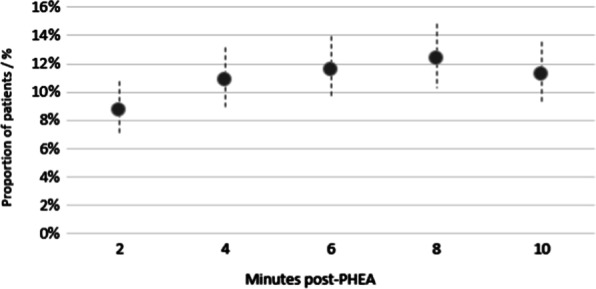
Point estimate chart showing the prevalence (cases at point in time) of hypotension in adult trauma patients who underwent PHEA in the East of England (defined as a new SBP < 90 mmHg ≤ 10 min of induction, or a > 10% drop if SBP was < 90 mmHg pre-PHEA) at two-minute epochs within the first ten minutes following induction. 95% Confidence Intervals, Wilson Score Method. PHEA prehospital emergency anaesthetic

218 (21.8%) patients had one or more new episode(s) of hypotension ≤ 10 min post-PHEA. Figure 2 shows the incidence of hypotension at two-minute intervals post-PHEA, defined as the first episode of hypotension for each patient. The peak incidence of PIH was at two minutes. Figure 3 shows the point prevalence of hypotension across the ten minutes, with a peak at eight minutes when 12.4% of patients had an episode of hypotension.

Table 2 shows the univariate association of the variables with the outcome. To test for plausible interactions in the regression model, interaction terms were fitted between variables and evaluated for significance. Where a statistically significant interaction was found, the fit of the model (based on likelihood ratio tests) was compared with and without the interaction term included. The model was a better fit in all cases without interaction terms, so none were reported in the final model.

**Table 2:** Univariate analysis: Association of variables with hypotension (defined as a new SBP < 90 mmHg ≤ 10 min of induction, or a > 10% drop if SBP was < 90 mmHg pre-PHEA) for adult trauma patients who underwent PHEA in the East of England, 2015–2020

Variable	Coefficient	*P*-value
Sex		
Male	REF	
Female	0.230	0.183*
Age/years		
16–34	REF	
35–54	0.120	0.547
55–74	0.349	0.084*
75 +	0.269	0.297
Estimated patient weight	− 0.002	0.691
GCS score	− 0.029	0.136*
Suspected injury pattern		
Isolated head injury	REF	
Head injury + thorax/abdomen	0.749	< 0.001*
No head injury	0.666	0.004*
Trauma type		
Blunt	REF	
Penetrating	0.100	0.809
Mechanism		
Transport	REF	
Accidental Injury	0.0817	0.630
Assault	− 0.354	0.375
Self-harm	0.611	0.075*
Sport/leisure	− 0.345	0.490
Shock index (SI)	0.818	< 0.001*
Pre-PHEA SBP/mmHg,		
Mid (90–140)	REF	
Low (< 90)	0.233	0.304
High (> 140)	− 0.998	< 0.001*
Pre-PHEA HR/beats/minute		
Mid (60–100)	REF	
Low (< 60)	0.326	0.268
High (> 100)	0.697	< 0.001*
Pre-PHEA RR/breaths/minute		
Mid (10–25)	REF	
High (> 25)	0.353	0.058*
Low (< 10)	0.071	0.837
PHEA drug regime (fentanyl:ketamine:rocuronium)		
3:2:1	REF	
1:1:1	0.687	0.036*
0:1:1	1.566	< 0.001*
0:0:1 (Roc only)	1.914	0.007*
Other	0.694	0.042*
Indication for PHEA		
Reduced consciousness	REF	
Airway obstruction/compromise	− 0.004	0.984
Ventilatory failure	0.523	0.017*
Agitated head injury	− 0.174	0.503
Anticipated clinical course	− 0.070	0.807
Other	− 13.250	0.976
Arrival time to PHEA/minutes	− 0.007	0.336
Pre-PHEA fluids		
None	REF	
Fluids	0.558	< 0.001*

The shock index was calculated as HR/SBP. ‘Arrival to PHEA’ is the time in minutes from the HEMS team arrival on scene until the time of PHEA. Pre-PHEA fluids are intravenous crystalloid administration by the ambulance service before arrival of HEMS

^*^P < 0.25 (threshold for including in first iteration of multivariate model)

*PHEA* prehospital emergency anaesthetic, *GCS* Glasgow Coma Scale, *RSI* rapid sequence induction, *HR* heart rate, *SBP* systolic blood pressure, *RR* respiratory rate

After the elimination of all non-significant variables and establishing the best-fit model, the final multivariate regression model was summarised, Table 3. Pre-PHEA SBP and HR were found to be strongly collinear (defined as a Variance Influence Factor > 5) with SI. Based on likelihood ratio tests, the model was a better fit including pre-PHEA SBP and HR separately rather than SI as a composite, so the latter was rejected. Only 12 records had missing data (pre-PHEA HR) in the final model, so imputation was not used. The McFadden’s pseudo-R-squared was 0.098.

**Table 3:** Final multivariate model-Adjusted association of variables with hypotension (defined as a new SBP < 90 mmHg ≤ 10 min of induction, or a > 10% drop if SBP was < 90 mmHg pre-PHEA) for adult trauma patients who underwent PHEA in the East of England, 2015–2020

Variable	Adjusted Odds ratio (95% CI)	*P*-value
Age/years		
16–34	REF	
35–54	1.23 (0.81–1.86)	0.324
55–74	2.13 (1.38–3.29)	< 0.001***
75 +	1.90 (1.08–3.31)	0.024 *
Pre-PHEA SBP/mmHg		
Mid (90–140)	REF	
Low < 90)	0.71 (0.43–1.15)	0.174
High (> 140)	0.37 (0.25–0.54)	< 0.001***
Pre-PHEA HR/beats/minute		
Mid (60–100)	REF	
Low (< 60)	1.43 (0.76–2.59)	0.256
High (> 100)	1.81 (1.28–2.57)	< 0.001***
RSI drug regime (fentanyl:ketamine:rocuronium)		
3:2:1	REF	
1:1:1	1.12 (0.68–1.83)	0.662
0:1:1	2.09 (1.29–3.41)	0.003**
0:0:1 (Roc only)	2.86 (1.34–6.09)	0.006**
Other	1.35 (0.81–2.24)	0.245
Pre-PHEA fluids		
None	REF	
Fluids	1.59 (1.13–2.23)	0.007**
Suspected injury pattern		
Isolated head injury	REF	
Head injury + thorax/abdomen	1.63 (1.13–2.36)	0.009**
No head injury	1.32 (0.80–2.15)	0.277

^*^P < 0.05, **P < 0.01, ***P < 0.001

*PHEA* prehospital emergency anaesthetic, *RSI* rapid sequence induction, *HR* heart rate, *SBP* systolic blood pressure, *RR* respiratory rate

Pre-PHEA fluids are intravenous crystalloid administration by the ambulance service before arrival of HEMS

### Primary outcome

Five variables were associated with PIH: patient age > 55 years old was associated with an increased hypotension risk, compared to those aged 16–34 years old; pre-PHEA heart rate of > 100 beats/minute was associated with hypotension, in contrast to a pre-PHEA SBP of > 140 mmHg, which was protective against the outcome; multi-system injuries (head injury with concomitant chest and/or abdominal injury), and intravenous crystalloid administration by the ambulance service before arrival of the HEMS team were also significantly associated with PIH. Drug regimes that omitted fentanyl (0:1:1 and 0:0:1 (rocuronium-only)) were the determinants with the largest effect sizes.

### Vasopressor administration

The multivariable regression model was re-run including administration of vasopressors within 10 min pre-or post-induction (*n* = 27), compared with the combined group of no vasopressor use, given outside of this time, or given but time not known. 17 (63.0%) were hypotensive after PHEA, and a further two patients (7.4%) were hypotensive at induction but their SBP did not drop by an additional 10%. In the adjusted multivariate regression model, the odds ratio for hypotension in the vasopressor group (compared with the baseline) was 5.25 (95%CI 2.26–12.84). Including vasopressor use in the model made no difference to the significance level of other variables and had negligible impact on effect sizes.

## Discussion

This study demonstrates that more than one in five patients who undergo PHEA have a new episode of significant hypotension within the first ten minutes of induction. Increasing patient age, multi-system injuries, a higher baseline heart rate, and intravenous crystalloid administration by the ambulance service before HEMS arrival were all significantly associated with PIH, whereas the addition of fentanyl to the induction drug regime was not.

Older people represent the fastest-growing proportion of society and the largest proportion of major trauma patients in England [24]. Consistent with UK trauma registry data, this study demonstrates that the highest proportion of patients who underwent PHEA were aged > 55 years old (*n* = *372*, 37.2%), affirming that major trauma is no longer a disease of the young [25]. The results identify an association between increasing age and the outcome, independent of injury burden or anaesthetic drug regime. Whilst this result is not surprising and is supported in the emergency airway literature, the doubling of PIH risk at an inflection point at 55 years old is younger than previously reported [26].

Major trauma patients are physiologically fragile, owing to a combination of insults such as haemorrhagic volume depletion, hypoperfusion, acidaemia, and hypoxaemia leading to reduced cardiac function and a reflex tachycardia. In this study, a baseline heart rate > 100 beats/minute was associated with PIH, in contrast to a baseline SBP > 140 mmHg that was protective. In addition, concomitant head, chest and/or abdominal injuries and intravenous crystalloid administration from the ambulance service before arrival of HEMS, were associated with PIH. In the UK, the ambulance service adheres to Joint Royal Colleges Ambulance Liaison Committee (JRCALC) guidance that advises cautious crystalloid administration in blunt polytrauma with *‘the aim of fluid therapy to maintain a palpable peripheral pulse or SBP* > *90 mmHg’* [27]. Whilst it is beyond the scope of this study to interrogate ambulance service physiological data, it is reasonable to assume that patients who were administered crystalloid before HEMS arrival are a physiologically compromised cohort.

Tachycardia and later hypotension are the normal physiological responses to haemorrhage [28]. This is frequently reported as the singular entity, ‘shock index’ (SI, a ratio of HR/SBP), demonstrated by Fouche et al.as a determinant of PIH, with a higher proportion of PIH observed in the cohort of patients with higher SI pre-induction [29]. The results of this study support these findings and that of Miller et al.who demonstrated that patients with an SI > 0.9 had a higher incidence of PIH than those < 0.9 [30]. Ketamine is considered a relatively cardiovascular stable drug and therefore recommended as the induction agent for critically ill patients [31]. The negative inotropic and vasodilatory effects in uninjured and haemodynamically-normal patients are weaker than the centrally mediated sympathomimetic effects [32]. The data in this study suggest that patients with potential catecholamine depletion and brain injury-induced autonomic dysfunction do not mount a sufficient sympathomimetic response, therefore the direct negative inotropic effect becomes dominant leading to significant hypotension [30, 33]. Consequently, PIH was observed more frequently in those with compromised baseline haemodynamics despite the use of a ‘cardiovascularly stable’ induction drug.

The addition of an opioid to the traditional RSI was introduced to attenuate the hypertensive response to laryngoscopy [7, 34]. Debate exists as to the potential negative haemodynamic effects of fentanyl [7, 17, 18, 20]. In this study, when fentanyl was omitted (0:1:1 and 0:0:1 (rocuronium only)) we observed the largest effect size of all variables with hypotension. Similar results were demonstrated by Ter Avest et al., who reported a signal of a larger proportion of PIH in patients who were administered a reduced-dose induction compared to a full dose [18]. There is no pharmacological basis on which to propose that large doses of fentanyl are conveying a ‘protective’ haemodynamic effect, and indeed there must be other variables at play that are not routinely recorded. This theory is supported by the Mcfadden’s pseudo-R-squared value of < 0.1 for the logistic regression model in this study. Whilst this is not equivalent to a linear regression R-squared and cannot strictly be interpreted as a ‘goodness of fit’ statistic, such a low value indicates that the variables captured in these data do not well-explain the variation in the outcome. However, clinician gestalt and provider intuition may be extremely accurate at identifying the patients most at risk of PIH, demonstrated by the choice of a reduced dose regime and the omission of fentanyl in this perceived high-risk patient group.

The aspiration at the outset of this study was to identify determinants of PIH and build a clinical decision model to reduce the risk of this outcome. Despite a large heterogenous dataset with excellent data completeness, it is clear from current data collection that this aspiration is not possible. What this study *has* been able to demonstrate is that clinician gestalt outperforms any other captured physiological variable or combination of variables for predicting prehospital PIH. Traditional data capture focuses on objective and binary physiological variables, rather than clinical intuition or learned experience, for example, the “Hateful Eight” clinical signs of haemorrhage that are recognised at scene but not translated into prehospital datasets [35]. If effective decision-support tools are to be developed, a shift is required from physiological data capture to a more nuanced approach incorporating the experiential and pragmatic components of complex clinical decision-making.

Most patients in this study sustained blunt traumatic head injuries without airway obstruction or ventilatory failure necessitating immediate airway intervention on the arrival of the HEMS team. Therefore, there is a potential window of opportunity to optimise patients before anaesthesia to avoid a deleterious haemodynamic insult. Proposed solutions that require further exploration and feasibility testing include the provision of invasive arterial monitoring to identify physiological trends earlier [36], volume loading with crystalloid or prehospital blood product before induction, a review of PHEA drug regimes, and peri-intubation vasopressor use [37, 38].

### Limitations

This study included the largest heterogeneous group of patients and clinicians exploring the haemodynamic effects of PHEA in the UK. Whilst this is a strength, it is possible that differences in practice may have influenced the results. Furthermore, these results report association and not causation, and therefore conclusions should be considered as hypothesis-generating.

The quality and completeness of data is often recognised as a challenge in prehospital academia, especially in retrospective analyses. However, missing data in physiological data capture work is less likely to be due to omissions in recording or documentation, and indeed more likely to reflect extreme physiological frailty, for example profound hypotension. In this study, 157 patients were excluded for missing data (mostly post-PHEA SBP), posing a risk of selection bias. These data are unlikely to be missing at random, and probably represent a cohort of patients with an unrecordable (low) blood pressure meeting the outcome definition. Therefore, this study may underestimate the true effect size.

## Conclusion

Patients > 55 years old; pre-PHEA tachycardia; multi-system injuries; and intravenous crystalloid administration before arrival of the HEMS team were the variables significantly associated with PIH. Induction drug regimes in which fentanyl was omitted (0:1:1 and 0:0:1 (rocuronium-only)) were the determinants with the largest effect sizes associated with hypotension. These variables only account for a small proportion of the observed outcome and clinician gestalt may play an important part.

## Data Availability

The datasets used and/or analysed during the current study are available from the corresponding author on reasonable request.

## Abbreviations

DBP: Diastolic blood pressure
EAAA: East Anglian Air Ambulance
EEAST: East of England Ambulance Service NHS Trust
EHAAT: Essex & Herts Air Ambulance
GCS: Glasgow Coma Scale
HEMS: Helicopter emergency medical service
HR: Heart rate
IQR: Interquartile range
JRCALC: Joint Royal Colleges Ambulance Liaison Committee
PEEP: Positive end-expiratory pressure
PHEA: Prehospital emergency anaesthesia
PIH: Post-intubation hypotension
RR: Respiratory rate
RSI: Rapid sequence induction
SBP: Systolic blood pressure
SD: Standard deviation
SI: Shock index
STROBE: Strengthening the Reporting of Observational studies in Epidemiology
UK: United Kingdom
